# Overexpression of Ras-Related C3 Botulinum Toxin Substrate 2 Radiosensitizes Melanoma Cells *In Vitro* and *In Vivo*

**DOI:** 10.1155/2019/5254798

**Published:** 2019-06-02

**Authors:** Wentao Hu, Lin Zhu, Weiwei Pei, Shuxian Pan, Ziyang Guo, Anqing Wu, Hailong Pei, Jing Nie, Bingyan Li, Yoshiya Furusawa, Teruaki Konishi, Tom K. Hei, Guangming Zhou

**Affiliations:** ^1^State Key Laboratory of Radiation Medicine and Protection, School of Radiation Medicine and Protection, Collaborative Innovation Center of Radiological Medicine of Jiangsu Higher Education Institutions, Soochow University, Suzhou 215123, China; ^2^Medical College of Soochow University, Suzhou 215123, China; ^3^Department of Basic Medical Sciences for Radiation Damages, National Institutes for Quantum and Radiological Science and Technology, Chiba 263-8555, Japan; ^4^Center for Radiological Research, College of Physician and Surgeons, Columbia University, New York, NY 10032, USA

## Abstract

Radioresistance is the major obstacle in the radiotherapy of the malignant melanoma. Thus, it is of importance to increase the radiosensitivity of melanoma cells. In the present study, the radioresistant melanoma cell line OCM-1 with inducible overexpression of Ras-related C3 botulinum toxin substrate 2 was established based on a radiation-inducible early growth response gene (Egr-1) promoter. The effects of Ras-related C3 botulinum toxin substrate 2 overexpression on the radiosensitivity of melanoma cells exposed to either X-rays or carbon ion beams were evaluated in cultured cells as well as xenograft tumor models. In addition, both reactive oxygen species yield and the NADPH oxidase activity were measured in the irradiated melanoma cells. It was found that the radiation-inducible overexpression of Ras-related C3 botulinum toxin substrate 2 sensitized the melanoma cells to both X-rays and carbon ion irradiation by enhancing the NADPH oxidase activity and the subsequent reactive oxygen species production. Besides, the overexpression of Ras-related C3 botulinum toxin substrate 2 enhanced the tumor-killing effect of radiotherapy in xenograft tumors significantly. The results of this study indicate that Ras-related C3 botulinum toxin substrate 2 is promising in increasing the radiosensitivity of melanoma cells, which provides experimental evidence and theoretical basis for clinical radiosensitization of the malignant melanoma.

## 1. Introduction

Melanoma, also known as malignant melanoma (MM), is a kind of malignant tumor originating from melanocytes in the basal layer of the epidermis. It has the characteristics of high degree of malignancy, early metastasis, poor prognosis, and high mortality. Radiotherapy is an important adjuvant therapy for advanced or metastatic melanoma. However, clinical trials indicate that melanoma is relatively resistant to conventional radiotherapy [1]. Therefore, it is of great significance to improve the radiosensitivity of melanoma cells for the treatment of this kind of malignancy. Carbon ion radiotherapy has achieved promising results in the treatment of MM in the past few years for the optimized dose distribution with the Bragg peak and the greater relative biologic effectiveness (RBE) [2]. However, due to the low 5-year survival rates in patients of advanced stages [3], the combination of gene therapy with carbon ion radiotherapy for improving the radiotherapy effect deserves further study.

The Ras-related C3 botulinum toxin substrate (RAC) protein family, as a member of the Rho GTP enzyme superfamily, has three subtypes in mammals: RAC1, RAC2, and RAC3, among which RAC1/2 is one of the cytosolic subunits of NADPH oxidase [4]. The NADPH oxidase, composed of 2 integral membrane proteins, p22^phox^ and gp91^phox^, and 4 cytosolic subunits: p47^phox^, p67^phox^, p40^phox^, and Rac1/2, is activated upon various stimuli and plays a central role in producing reactive oxygen species (ROS) *via* transferring electrons from cytosolic NADPH to O_2_ [5]. Compared with RAC1, RAC2 has a higher affinity with NADPH oxidase; thus, it can promote the production of ROS more effectively [6, 7]. With the key role in the regulation of the activity of NADPH oxidase, RAC2 has been found to be involved in the regulation of a diverse array of cellular events, including cell growth, inflammation, chemotaxis, cell polarization, cell adhesion, and macrophage activation [8–13]. It is also reported that functional disruption of RAC2 caused severe myeloid cell dysfunction in both mouse and human [14]. As is known, ROS of normal physiological level is necessary to host defense and cellular signal transduction. However, excessive ROS will lead to oxidative stress, cell dysfunction, and even apoptosis or necrosis [15]. Many studies have shown that ROS produced by NADPH oxidase is an important mediator for radiobiological effects and is also the decisive factor of cellular radiosensitivity [16–18]. It has been found that the ROS production is tightly associated with the radioresistance of cancer stem cells and removal of ROS scavengers sensitized the cancer stem cells to radiation [19, 20]. Basing on these findings, we speculate that RAC2 is likely to regulate the radiosensitivity of the tumor cells by modulating the activity of NADPH oxidase.

Although RAC2 has been studied for the regulation of activity of NADPH oxidase, the studies of the relationship between RAC2 and radiosensitivity of tumor cells are limited. Watanabe et al. used microarray to study the gene expression of tissue samples from rectal cancer patients who received preoperative radiotherapy. It was found that RAC2 expression in nonresponders was significantly lower than that in responders, but the underlying mechanism is not clear [21]. In the previous work, we studied the radiosensitivity of several melanoma cell lines and found that 92-1 was more radiosensitive, while OCM-1 presented radiation resistance [22]. In this study, we chose the melanoma cell lines 92-1 and OCM-1 of different radiosensitivity as a set of experimental models based on the results of previous experiments, for the purpose of revealing the effects and underlying mechanisms of RAC2 on the radiosensitivity of melanoma cells both *in vitro* and *in vivo*.

## 2. Materials and Methods

### 2.1. Cell Culture and Irradiation

Human MM cell lines 92-1 and OCM-1 are kind gifts from Professor Shengfang Ge of the Ninth People's Hospital Affiliated to Shanghai Jiaotong University School of Medicine. Cells were maintained in Dulbecco's modified Eagle's medium (DMEM; Gibco, Grand Island, NY, USA) supplemented with 10% fetal bovine serum (FBS; Gibco, Grand Island, NY, USA), 1% penicillin sodium, and 100 *μ*g/mL streptomycin, at 37°C in 5% CO_2_ in a humidified incubator (Thermo Fisher Scientific, NC, USA). The 92-shRAC2 and OCM-RAC2, with RAC2 knockdown in 92-1 cells and radiation-inducible RAC2 overexpression in OCM-1 cells, respectively, as well as their negative control cell lines (92-NC and OCM-NC) were established by corresponding lentivirus infection and puromycin selection. The lentivirus for RAC2 knockdown or overexpression was designed and packed by Sangon Biotech (Shanghai, China) (Fig.  S1 and S2). The overexpression of RAC2 was verified by Western blot analysis (Fig.  S3). X-ray irradiation was performed at the State Key Laboratory of Radiation Medicine and Protection, Soochow University, by using a RS 2000 X-ray Biological Irradiator (Rad Source Technologies, Suwanee, GA, USA) at a dose rate of 1.2 Gy/min. Carbon ion irradiation was performed at Heavy-Ion Medical Accelerator in Chiba (HIMAC) at the National Institute of Radiological Sciences (NIRS), Chiba, Japan, with 290 MeV/n carbon ions. The LET value for carbon ions was 13.3 keV/*μ*m. The dose rate was 1 Gy/min.

### 2.2. Clonogenic Survival Assay

The clonogenic survival assay was conducted as previously described [23]. Briefly, cells were harvested immediately after irradiation by trypsinization, then counted, and plated into Φ60 mm dishes and returned to the incubator to produce 20-100 clones. After growing for 14 days, cells were fixed with 70% ethanol for 5 min and stained with 0.5% crystal violet. Colonies containing more than 50 cells were counted as survivors. At least 3 parallel dishes were scored for each treatment.

### 2.3. Western Blotting

Cells were lysed using RIPA buffer (Beyotime, Nantong, China). Samples were sonicated and centrifuged at 12000 rpm for 15 min at 4°C. The total protein concentration was determined by using a DC Protein Assay Kit (Bio-Rad, Richmond, CA, USA). Samples were denatured at 100°C for 5 min, separated using SDS-PAGE, and transferred to polyvinylidene difluoride (PVDF) membranes (GE Healthcare, Piscataway, NJ, USA). After blockage with PBST (PBS with 0.1% Tween-20) containing 5% skimmed milk for 1 h, the membrane was incubated with primary antibodies for 2 h at room temperature, washed three times with PBST for 5 min each, and then incubated with HRP-conjugated secondary antibody for 1 h and washed three times with PBST. Protein bands were visualized using an ECL kit (Millipore, Billerica, MA, USA) Anti-RAC2 was purchased from Abcam (Cambridge, MA, USA), and anti-GAPDH was from Cell Signaling Technology (Beverly, MA, USA).

### 2.4. Measurement of ROS Yield and NADPH Oxidase Activity

ROS yield and NADPH oxidase activity of the treated cells were measured using the ROS Detection Kit (Beyotime, Nantong, China) and the NADPH Oxidase Assay Kit (Jiancheng, Nanjing, China) in strict accordance with the manufacturers' manuals. The corresponding absorbance was detected on a multidetection microplate reader (Synergy2; BioTek Instruments, VT, USA).

### 2.5. Immunofluorescence

Cells were grown on coverslips in 12-well plates. After irradiation, cells were fixed with 4% formaldehyde in PBS at room temperature for 10 min and methanol at -20°C for 20 min, permeabilized in 0.1% Triton X-100 in PBS for 10 minutes, and blocked with 5% skim milk for 1 h. Cells were then incubated at room temperature for 2 hours with anti-*γ*H2AX (Cell Signaling Technology, Beverly, MA, USA). Cells were then stained with Alexa Fluor® 555 anti-rabbit antibody (Molecular Probes, Eugene, OR, USA) at 37°C for 1 hour. Following extensive washing in PBS, the cells were mounted on slides using DAPI mounting medium (Eugene, OR, USA). The stained cells were observed under an Olympus IX71 microscope (Olympus, Tokyo, Japan) in NIRS while under a laser scanning confocal microscope (Olympus FV1200, Tokyo, Japan) in Soochow University. At least 100 cells were scored for each sample.

### 2.6. Micronucleus and Apoptosis Assay

Cells were seeded in 12-well plates. Immediately after 2 Gy X-rays or carbon ion beam irradiation, cells were incubated with medium containing 2.5 *μ*g/mL cytochalasin B (Sigma-Aldrich, St. Louis, MO, USA) for the micronucleus (MN) assay. Thirty hours later, cells were washed with PBS and fixed with Carnoy's solution for 15 min. After being stained with 30 *μ*g/mL acridine orange (Sigma-Aldrich, St. Louis, MO, USA), at least 500 binucleated cells for each sample were counted for micronucleus frequency (MNF) calculation. For apoptosis assays, cells were harvested 48 hours post 4 Gy X-rays or carbon ion beam irradiation and stained using Annexin-V-APC/PI kit (BD Biosciences, San Jose, CA, USA) according to the manufacturer's instructions and analyzed by a FACSVerse flow cytometer (BD Biosciences, Franklin Lakes, NJ, USA).

### 2.7. Xenograft Studies

10^6^ OCM-NC or OCM-RAC2 cells were respectively injected subcutaneously into the flanks of 6-week-old male nude mice (Slaccas, Shanghai, China). Three weeks later, all mice developed solid tumors with a volume of about 100 mm^3^. The tumors were irradiated by a cone-beam CT-guided precision irradiation system (X-RAD 225Cx; Precision X-Ray, North Branford, CT, USA) at a single dose of 8 Gy after mice were anesthetized by sodium pentobarbital (40 mg/kg intraperitoneally). Then, the tumor volumes were measured every 5 d using a caliper for 1 month, and the tumor volumes were calculated using the formula *V* = *ab* ^2^ × *π*/6, where *a* is the length and *b* is the width. All tumor volume data were normalized to those obtained just before irradiation. One month after irradiation, the mice were sacrificed and their tumors were collected for weighing, paraffin section preparation as well as immunohistochemical staining. All mice were maintained in the SPF Animal Laboratory of Soochow University. All animal studies were reviewed and approved by the Soochow University Institutional Animal Care and Use Committee.

### 2.8. Statistical Analysis

Statistical analysis was performed on the means of the data obtained from at least 3 independent experiments. Data are presented as the means ± SE. *p* values between the indicated samples were additionally presented. *p* < 0.05 was considered to be statistically significant.

## 3. Results

### 3.1. Effects of RAC2 on Colony Forming of Irradiated Melanoma Cells

**Figure 1:**
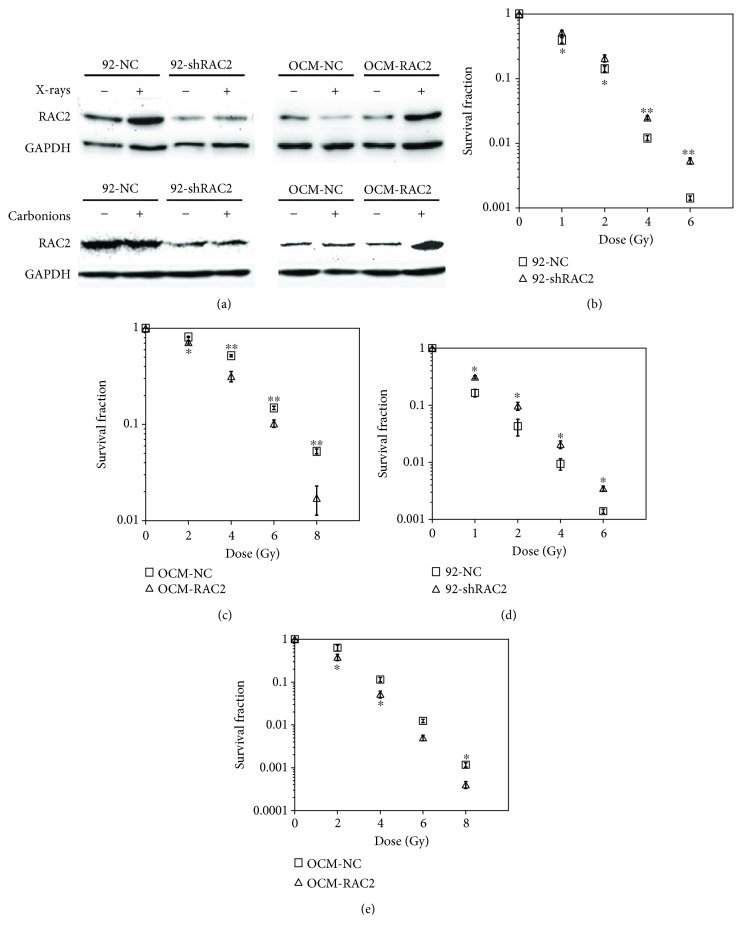
The RAC2 expression levels and the survival fraction of the 4 cell lines exposed to X-rays or carbon ion beams. (a) The RAC2 protein levels in the 4 cell lines exposed to 2 Gy X-rays or 2 Gy carbon ion beams with RAC2 knockdown or radiation-inducible RAC2 overexpression were detected by Western blot 2 hours postirradiation. (b, c) After exposure to different doses of X-rays, the survival fractions of the 4 cell lines were measured by colony forming assay. (d, e) After exposure to different doses of carbon ion beams, the survival fractions of the 4 cell lines were measured by colony forming assay. ^∗^ indicates a statistical significance (*p* < 0.05) for comparisons between 92-NC and 92-shRAC2 or between OCM-NC and OCM-RAC2. ^∗∗^ *p* < 0.01.

We detected RAC2 expression in several melanoma cell lines and found that RAC2 expression in 92-1 cells was much higher than that in OCM-1 cells (Fig.  S4). Then, 92-shRAC2 and OCM-RAC2, with RAC2 knock-down in 92-1 cells and radiation-inducible RAC2 overexpression in OCM-1 cells respectively, as well as their negative control cell lines (92-NC and OCM-NC) were established by corresponding lentivirus infection and puromycin selection. The expressions of RAC2 in 4 cell lines after X-ray or carbon ion irradiation were verified by Western blot. As shown in Figure 1(a), RAC2 expression was increased in X-ray-irradiated 92-NC cells while decreased in X-ray-irradiated OCM-NC cells while no significant change was observed upon carbon ion irradiation in both cell lines. However, RAC2 expression was decreased significantly in 92-shRAC2 cells while increasing markedly in OCM-RAC2 cells exposed to either X-rays or carbon ions. Then, a colony forming assay was employed to examine cell survival fraction following irradiation. Compared with the control group, RAC2 knockdown resulted in a significantly higher survival rate of 92-1 cells exposed to either X-ray or carbon ion irradiation (Figures 1(b) and 1(d)), while radiation-inducible RAC2 overexpression significantly radiosensitized OCM-1 cells to both X-rays and carbon ions (Figures 1(c) and 1(e)). These results indicate that RAC2 is positively correlated with the radiosensitivity of melanoma cells.

### 3.2. Effects of RAC2 on ROS Yield and Activity of NADPH Oxidase in Irradiated Melanoma Cells

**Figure 2:**
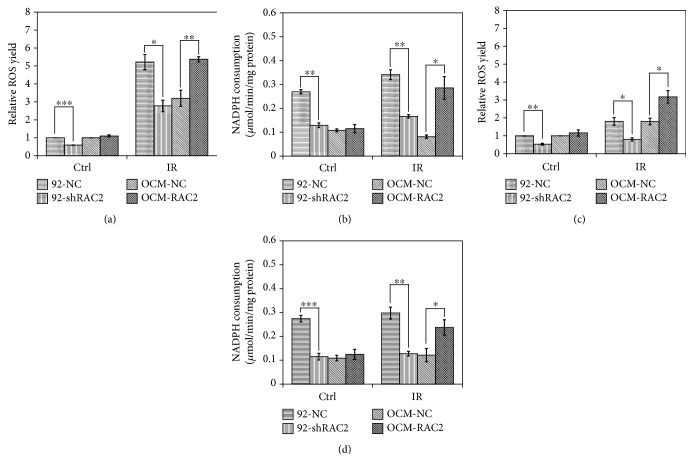
The effects of RAC2 on the ROS yield and NADPH oxidase activity of the 4 cell lines exposed to 2 Gy X-rays or 2 Gy carbon ion beams. (a) The ROS yields in the 4 cell lines were measured after X-ray exposure. (b) NADPH consumption in the 4 cell lines after X-ray exposure. (c) The ROS yields in the 4 cell lines were measured after carbon ion beam exposure. (d) NADPH consumption in the 4 cell lines after carbon ion beam exposure. ^∗^ *p* < 0.05, ^∗∗^ *p* < 0.01, and ^∗∗∗^ *p* < 0.001.

**Figure 3:**
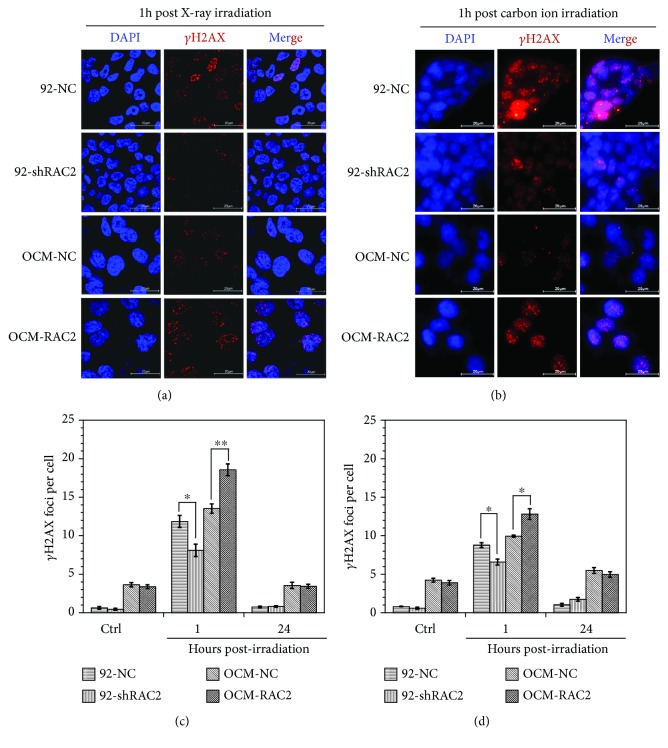
The *γ*H2AX levels in the 4 cell lines exposed to 0.5 Gy X-rays or 0.5 Gy carbon ion beams. (a) Immunofluorescent staining of the *γ*H2AX in the cells exposed to 0.5 Gy X-rays. (b) Immunofluorescent staining of the *γ*H2AX in the cells exposed to 0.5 Gy carbon ion beams. (c) The *γ*H2AX yields in the cells after X-ray exposure. (d) The *γ*H2AX yields in the cells after carbon ion beam exposure. ^∗^ *p* < 0.05 and ^∗∗^ *p* < 0.01.

Since RAC2 is a key factor in the regulation of NADPH oxidase activity and the production of reactive oxygen species (ROS) and ROS is decisive in radiation-induced cell death, the intracellular ROS production and NADPH oxidase activity 0.5 h after 2 Gy X-rays or carbon ion beams were measured in the established cell lines. Both X-ray irradiation-induced ROS production and the activity of NADPH oxidase were significantly suppressed in 92-shRAC2 cells compared with 92-NC cells. Besides, the X-ray irradiation-induced ROS production as well as NADPH oxidase activity was significantly enhanced in OCM-RAC2 cells compared with OCM-NC cells (Figures 2(a) and 2(b)). Similar results were obtained in the 4 cell lines exposed to carbon ions (Figures 2(c) and 3(d)). These results indicate that overexpressed RAC2 activates NADPH oxidase and promotes ROS production in OCM-1 cells.

### 3.3. The Effects of RAC2 on *γ*H2AX Formation after Irradiation

Since the radiation-induced DNA double-strand breaks (DSB) are a leading cause of cell death, the *γ*H2AX focus levels in the established cell lines after exposure to X-ray or carbon ion irradiation were measured to evaluate the effects of RAC2 expression on DSB induction after irradiation. As shown in Figure 3, the *γ*H2AX focus level in 92-shRAC2 cells was significantly lower than that of the control group 1 hour after 0.5 Gy X-ray irradiation, while the *γ*H2AX focus level in OCM-RAC2 cells was significantly higher than that of OCM-NC (Figures 3(a) and 3(c)). Similar results were also observed in the 4 cell lines exposed to 0.5 Gy carbon ion beam irradiation even though the *γ*H2AX focus levels were much lower in carbon ion-irradiated cells (Figures 3(b) and 3(d)). These results indicate that RAC2 aggravates the DSB formation in melanoma cells after X-ray or carbon ion irradiation, further demonstrating the radiosensitizing effect of RAC2 on melanoma cells.

### 3.4. Effects of RAC2 on Irradiation-Induced Micronucleus Formation and Apoptosis

**Figure 4:**
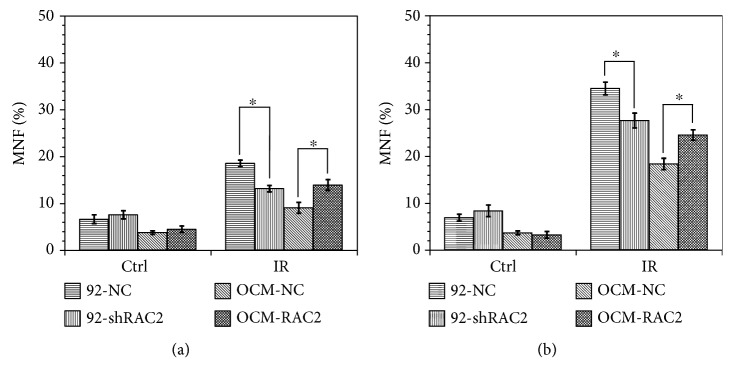
The micronucleus frequency in the 4 cell lines exposed to 2 Gy X-rays or 2 Gy carbon ion beams. (a) The micronucleus frequency in the cells exposed to 2 Gy X-rays. (b) The micronucleus frequency in the cells exposed to 2 Gy carbon ion beams. ^∗^ *p* < 0.05.

**Figure 5:**
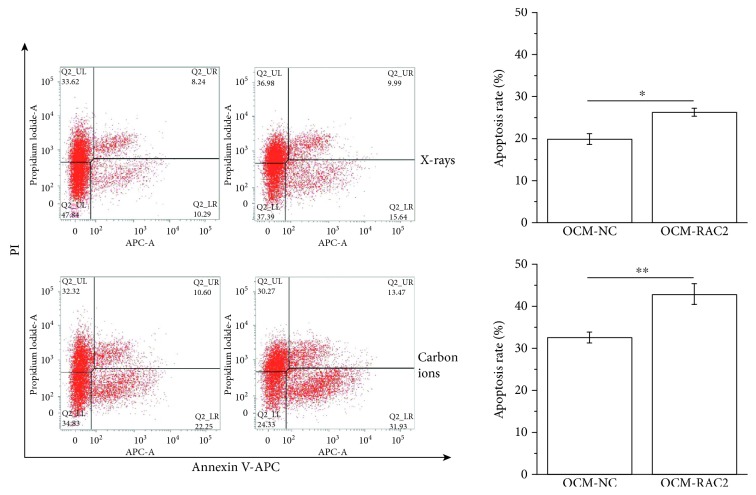
Overexpression of RAC2 enhanced radiation-induced apoptosis of melanoma cells. OCM-NC or OCM-RAC2 cells were irradiated with 4 Gy X-rays or 4 Gy carbon ion beams. After 48 hours, cells were collected and stained with Annexin V-APC/PI and then analyzed using flow cytometry. Representative images and quantitative data are shown. ^∗^ *p* < 0.05 and ^∗∗^ *p* < 0.01.

Micronucleus formation is recognized as an important characterization of radiation-induced chromosome damage. The micronucleus frequency (MNF) in 92-shRAC2 cells was significantly lower than that in 92-NC cells after 2 Gy X-ray or 2 Gy carbon ion irradiation, but no difference was observed between control groups. Correspondingly, the MNF in OCM-RAC2 cells was significantly higher than that in OCM-NC cells after 2 Gy X-ray or 2 Gy carbon ion irradiation, while no difference between control groups, either (Figures 4(a) and 4(b)). We also measured radiation-induced apoptosis in OCM-NC and OCM-RAC2 cells and found that X-ray or carbon ion irradiation-induced RAC2 overexpression combined with radiation resulted in significantly higher apoptosis rate in OCM-RAC2 cells (Figure 5), while inhibition of NADPH oxidase activity by 10 *μ*M DPI abolished the radiosensitizing effect induced by RAC2 overexpression (Fig.  S5). These results indicate that radiation-induced RAC2 overexpression promoted X-ray or carbon ion irradiation-induced micronucleus formation and apoptosis in melanoma cells.

### 3.5. Enhancement of Melanoma Cell Radiosensitivity *In Vivo* by RAC2 Overexpression

**Figure 6:**
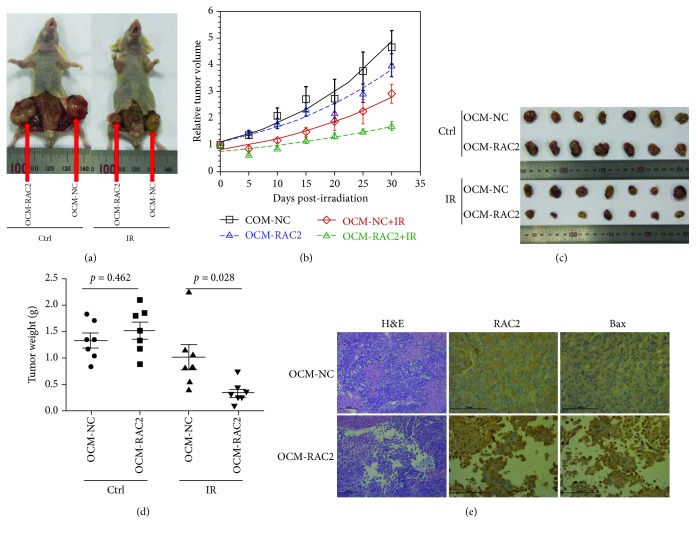
Overexpression of RAC2 enhanced melanoma cell radiosensitivity *in vivo*. (a) Representative image of tumors generated by OCM-NC or OCM-RAC2 cells in nude mice 1 month post 8 Gy X-ray irradiation. (b) Growth curves of OCM-NC and OCM-RAC2 tumors (*n* = 8). (c) Xenografted tumors collected for weighing. (d) The weights of the xenografted tumors from different groups were compared. (e) H&E staining of the xenografted tunors as well as IHC staining of RAC2 and Bax in xenografted tumors.

To explore the effects of RAC2 overexpression on the radiosensitivity of melanoma cells *in vivo*, xenograft tumor models were established by injecting OCM-NC and OCM-RAC2 cells subcutaneously into the flanks of 6-week-old nude mice (*n* = 8 flanks). Radiation treatment of 8 Gy X-rays was conducted after the tumor volume reached about 100 mm^3^. Then, tumor sizes were measured every 5 days postirradiation with a caliper to calculate tumor volumes (Figure 6(b)). One month later, all mice were sacrificed and the tumors were collected for weighing and immunohistochemical (IHC) analysis of RAC2 and Bax expression. As shown in Figures 6(a), 6(c), and 6(d), the tumor sizes of the OCM-RAC2 group were much larger than those of the OCM-NC group when exposed to the same dose of irradiation. H&E staining of the tumor sections showed that irradiation triggered more cellular atrophy in the tumors derived from OCM-RAC2 cells compared with that from OCM-NC cells, while IHC analysis showed that the RAC2 as well as Bax expression in tumors derived from OCM-RAC2 cells was much higher than that from OCM-NC tumors (Figure 6(e)).

## 4. Discussion

Radioresistance is one of the major obstacles in the radiotherapy of malignant melanoma [1]. Thus, it is important to develop radiosensitizing methods to improve the radiotherapy and decrease its side effects. Better therapeutic effects have been achieved for this kind of malignancy by taking use of heavy ion beams, which are characterized by higher biological effectiveness and lower oxygen effect compared with photons [24]. In the present work, we proposed to study the radiosensitizing effect of RAC2 on melanoma cells to further improve the treatment effects. We compared the expression of RAC2 in 2 human melanoma cell lines of different radiosensitivity and found that the expression of RAC2 is positively correlated with the radiosensitivity of melanoma cells. By further research, we found that RAC2 can promote the ROS production by activating NADPH oxidase, as well as increase the cellular radiosensitivity by aggravating radiation-induced DNA damage. Besides, the increased radiosensitivity of OCM-1 cells may also correlate with the aggravated cell cycle arrest in the G2/M phase induced by elevated ROS production (Fig.  S6). The results are consistent with our previous findings in quiescent human embryonic lung fibroblasts [25].

RAC2, as a key subunit of NADPH oxidase complex, functions in activating NADPH oxidase and promoting the production of ROS [26]. ROS is the mediator and major contributor of DNA damage induced by low LET ionizing radiation and is the decisive molecule of radiation-induced cell death. However, there has not been a report of application of RAC2 in tumor radiosensitization. The results of this study are of great significance to clarify the regulatory mechanisms of RAC2 on the radiosensitivity of melanoma cells and provide an important theoretical basis for the development of clinical radiosensitization methods based on the RAC2 gene. As shown in the results, although carbon ion irradiation did not change the expression of RAC2 in either 92-1 or OCM-1 cells, the knockdown or overexpression of RAC2 did influence the radiosensitivity by modulating the activity of NADPH oxidase.

Compared with the conventional photon radiations, the energy of heavy ion radiation is precisely deposited in the tumor site due to its unique inverted depth-dose distribution, which can not only enhance the tumor-killing ability but also effectively reduce the damage of the surrounding healthy tissue and reduce complications. Carbon ion irradiation has been shown to be effective in the treatment of cutaneous melanoma [27], mucosal melanoma [28], and ocular melanoma [29]. However, as is known, heavy ion irradiation-induced DNA damage arises mainly as a consequence of the direct interaction of the ionizing particles with the DNA molecules [30, 31]. It remains unclear whether the cell-killing effects of the heavy ion irradiation could be enhanced by combination with the indirect effects through increasing of endogenous ROS production. In the current study, it was found that upon radiation-inducible RAC2 overexpression, which led to the increase of NADPH oxidase activity, the endogenous ROS production was promoted and the radiation-induced decrease of the survival fraction of melanoma OCM-1 cells was aggravated significantly. However, both OCM-1 cells and 92-1 cells we used in this study are cell lines of ocular melanoma. As is known, the prognosis of the ocular melanoma is not so poor as the malignant melanoma of other sites. In order to generalize the application of our results, it is necessary to expand our findings to the melanoma cell lines of other sites.

## 5. Conclusions

In conclusion, RAC2 was found to play a role in the radiosensitization of human malignant melanoma cells by activating NADPH oxidase and facilitating the ROS production. Whether RAC2 could be used in the radiosensitization of other kinds of tumors requires further study. In addition, since RAC2 is involved in multiple signaling pathways, the side effects of RAC2 overexpression need further investigation.

## Data Availability

The data used to support the findings of this study are available from the corresponding authors upon request.

## Abbreviations

RAC2: Ras-related C3 botulinum toxin substrate 2
MM: Malignant melanoma
RBE: Relative biologic effectiveness
NADPH: Nicotinamide adenine dinucleotide phosphate
ROS: Reactive oxygen species
LET: Linear energy transfer
MNF: Micronucleus frequency
DSB: DNA double-strand breaks
IHC: Immunohistochemistry.

## Supplementary Materials

Supplementary Materials Figure S1: the sketch map of the plasmid (A) and the sequences of shRNA (B) used for RAC2 knockdown. Figure S2: the sketch map of the plasmid (A) and the sequence of Egr1-RAC2 (B) used for RAC2 overexpression, in which the Egr1 sequence was shown in gray and RAC2 in yellow while Flag in purple. Figure S3: overexpression of RAC2 in OCM-RAC2 cells postirradiation was verified by Western blot analysis of Flag at indicated time points. Figure S4: relative RAC2 expressions in several melanoma cell lines. Figure S5: inhibition of NADPH oxidase activity by 10 *μ*M DPI abolished the radiosensitizing effect induced by RAC2 overexpression. Figure S6: cell cycle distribution of the 4 cell lines exposed to 2 Gy X-ray irradiation.
