# Supplementary material for: Overexpression of Ras-Related C3 Botulinum Toxin Substrate 2 Radiosensitizes Melanoma Cells In Vitro and In Vivo
Source: Oxid Med Cell Longev. 2019 Jun 2;2019:5254798. doi: 10.1155/2019/5254798 (PMC6589259; doi:10.1155/2019/5254798)
Supplement: Supplementary Materials — Figure S1: the sketch map of the plasmid (A) and the sequences of shRNA (B) used for RAC2 knockdown. Figure S2: the sketch map of the plasmid (A) and the sequence of Egr1-RAC2 (B) used for RAC2 overexpression, in which the Egr1 sequence was shown in gray and RAC2 in yellow while Flag in purple. Figure S3: overexpression of RAC2 in OCM-RAC2 cells postirradiation was verified by Western blot analysis of Flag at indicated time points. Figure S4: relative RAC2 expressions in several melanoma cell lines. Figure S5: inhibition of NADPH oxidase activity by 10 μM DPI abolished the radiosensitizing effect induced by RAC2 overexpression. Figure S6: cell cycle distribution of the 4 cell lines exposed to 2 Gy X-ray irradiation. [file 5254798.f1.pptx]

## Slide 1
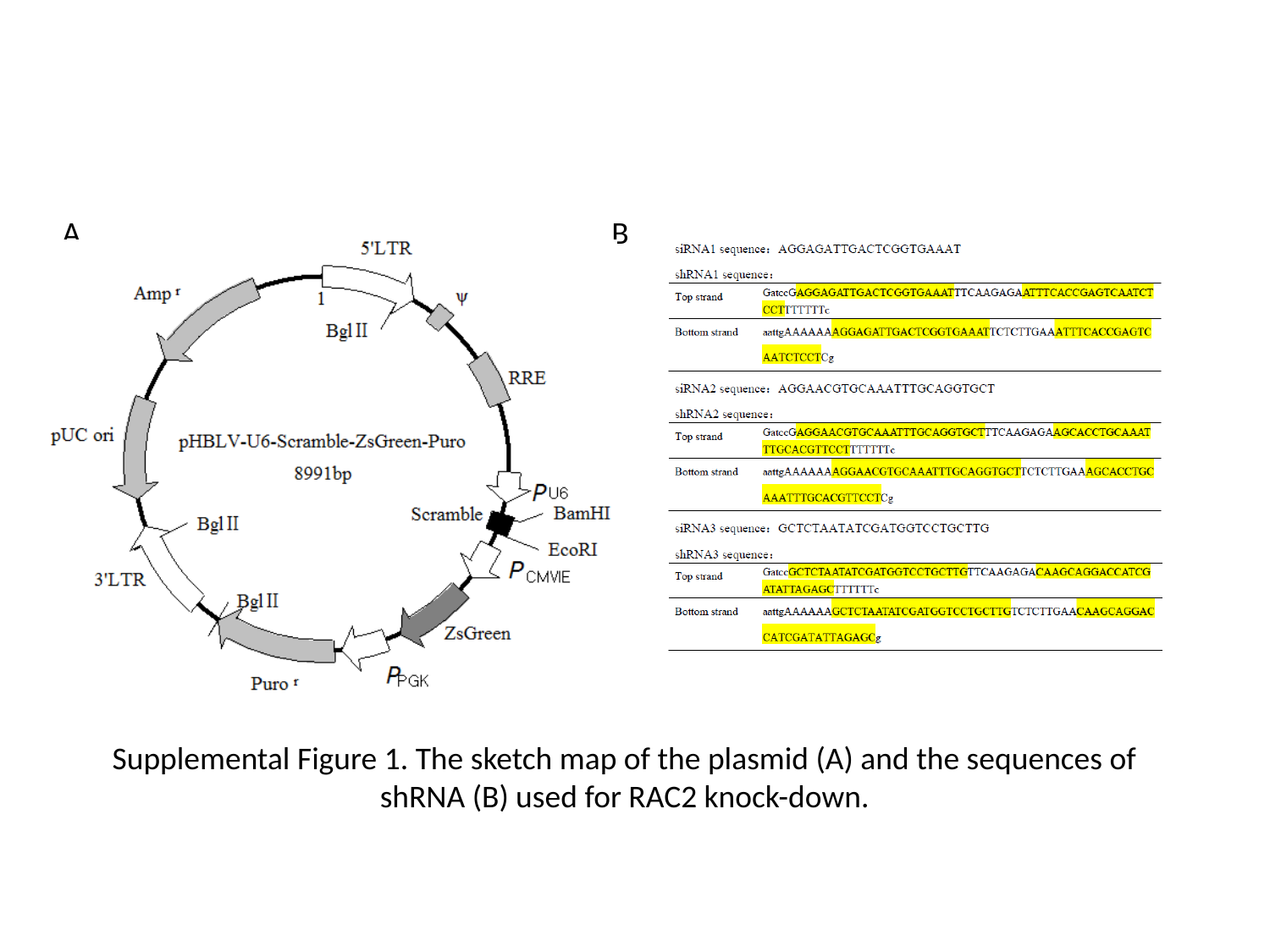

A B
Supplemental Figure 1. The sketch map of the plasmid (A) and the sequences of shRNA (B) used for RAC2 knock-down.

## Slide 2
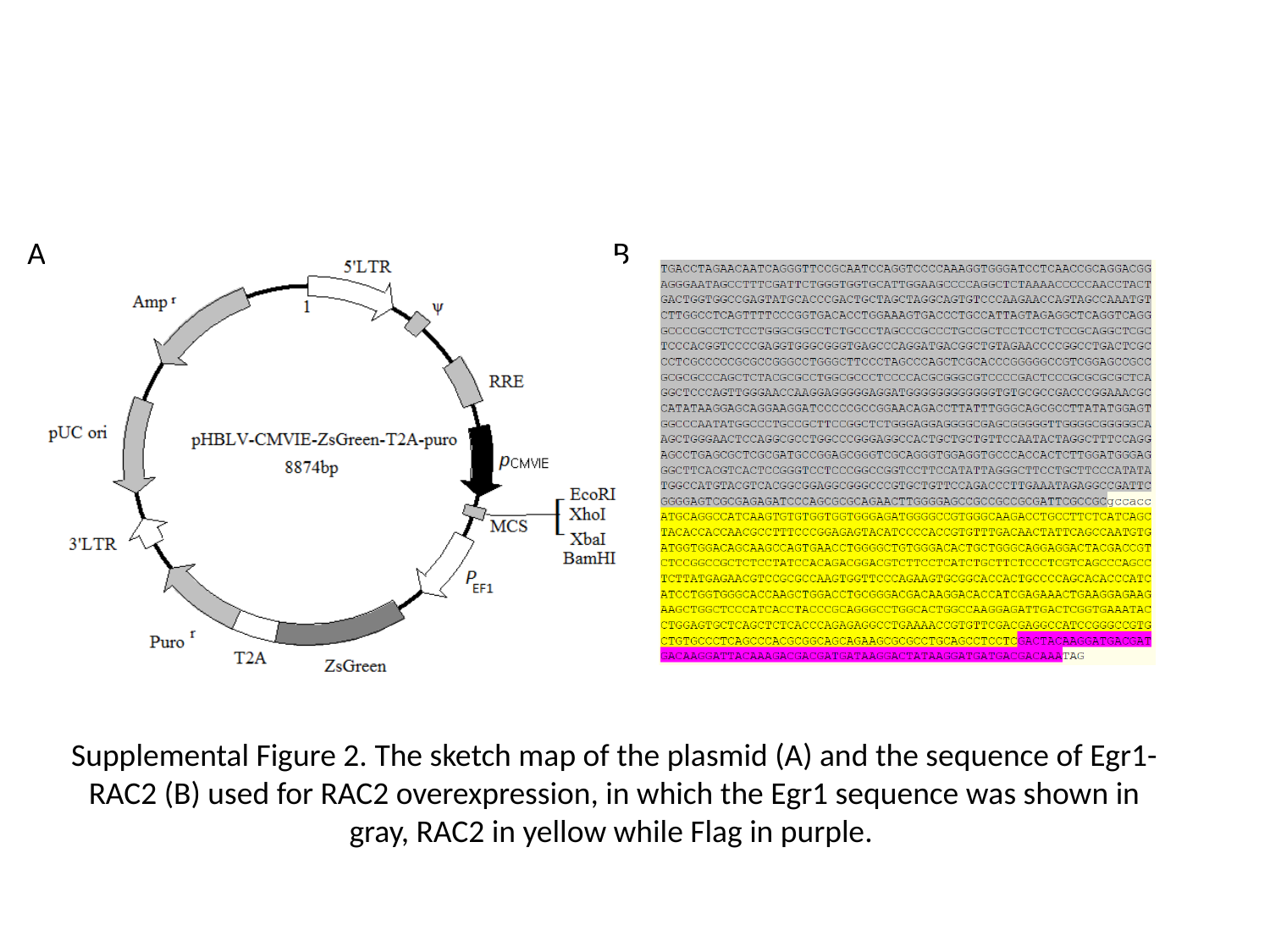

A B
Supplemental Figure 2. The sketch map of the plasmid (A) and the sequence of Egr1-RAC2 (B) used for RAC2 overexpression, in which the Egr1 sequence was shown in gray, RAC2 in yellow while Flag in purple.

## Slide 3
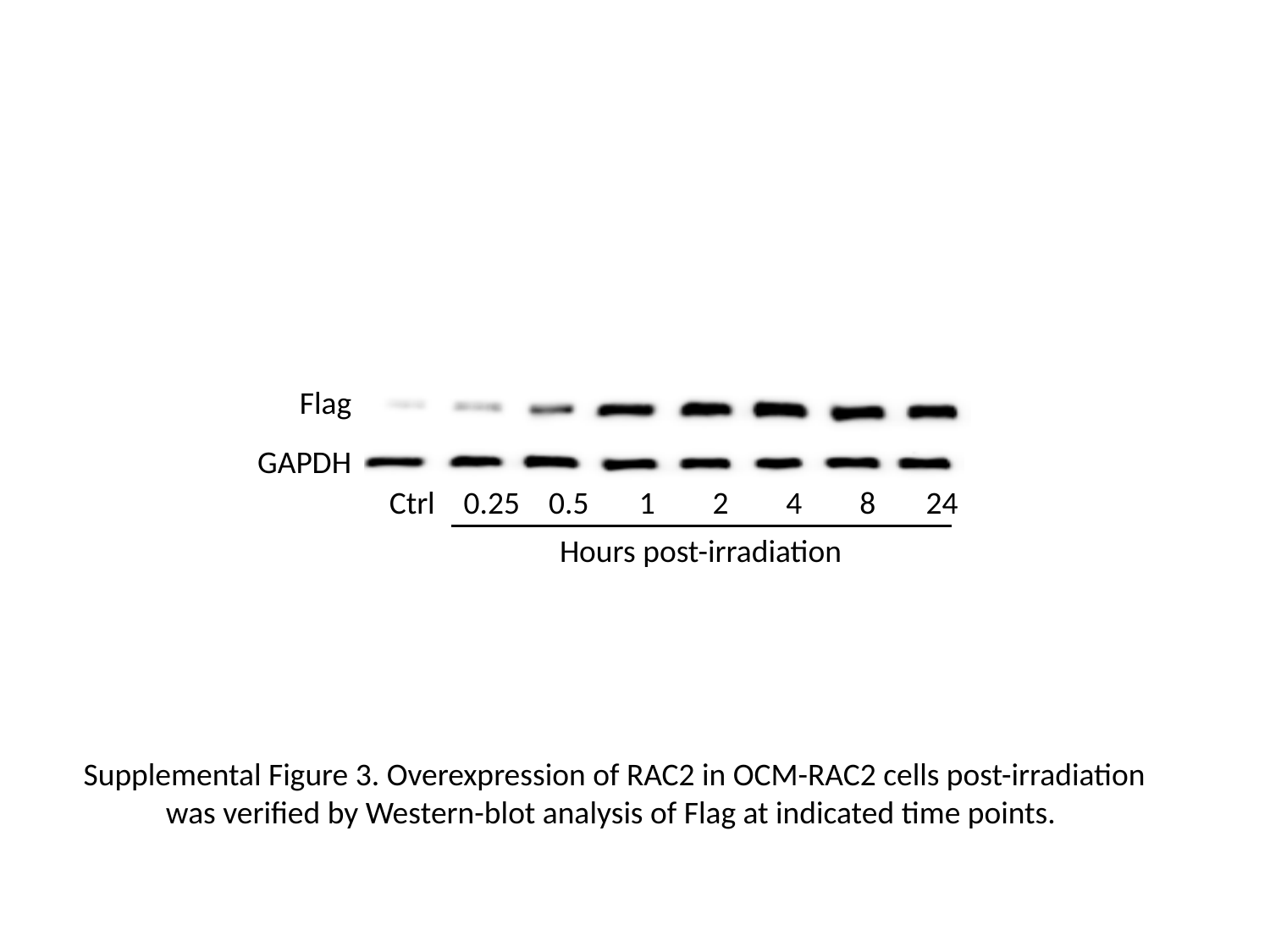

Flag
GAPDH
Ctrl 0.25 0.5 1 2 4 8 24
Hours post-irradiation
Supplemental Figure 3. Overexpression of RAC2 in OCM-RAC2 cells post-irradiation was verified by Western-blot analysis of Flag at indicated time points.

## Slide 4
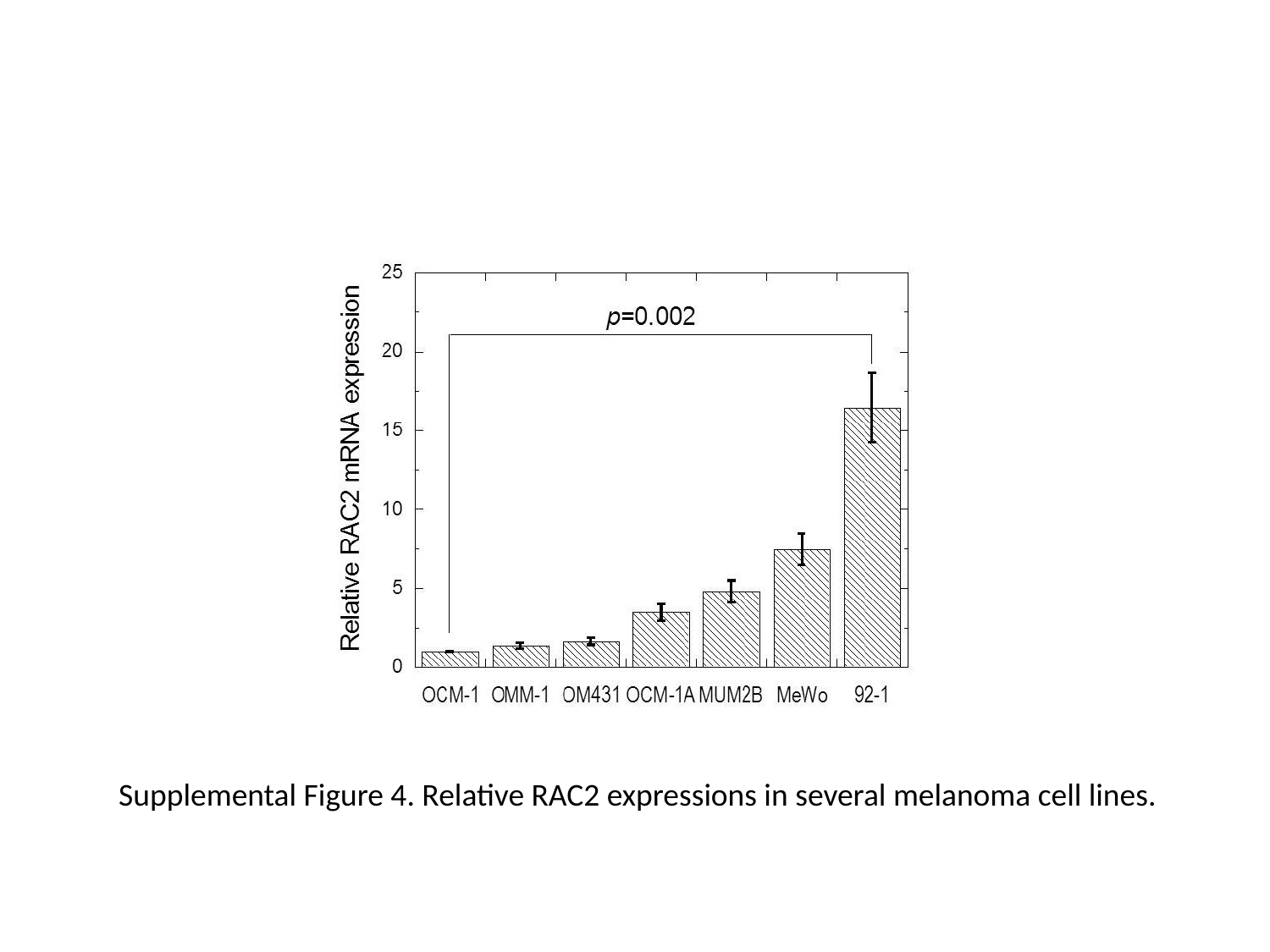

Supplemental Figure 4. Relative RAC2 expressions in several melanoma cell lines.

## Slide 5
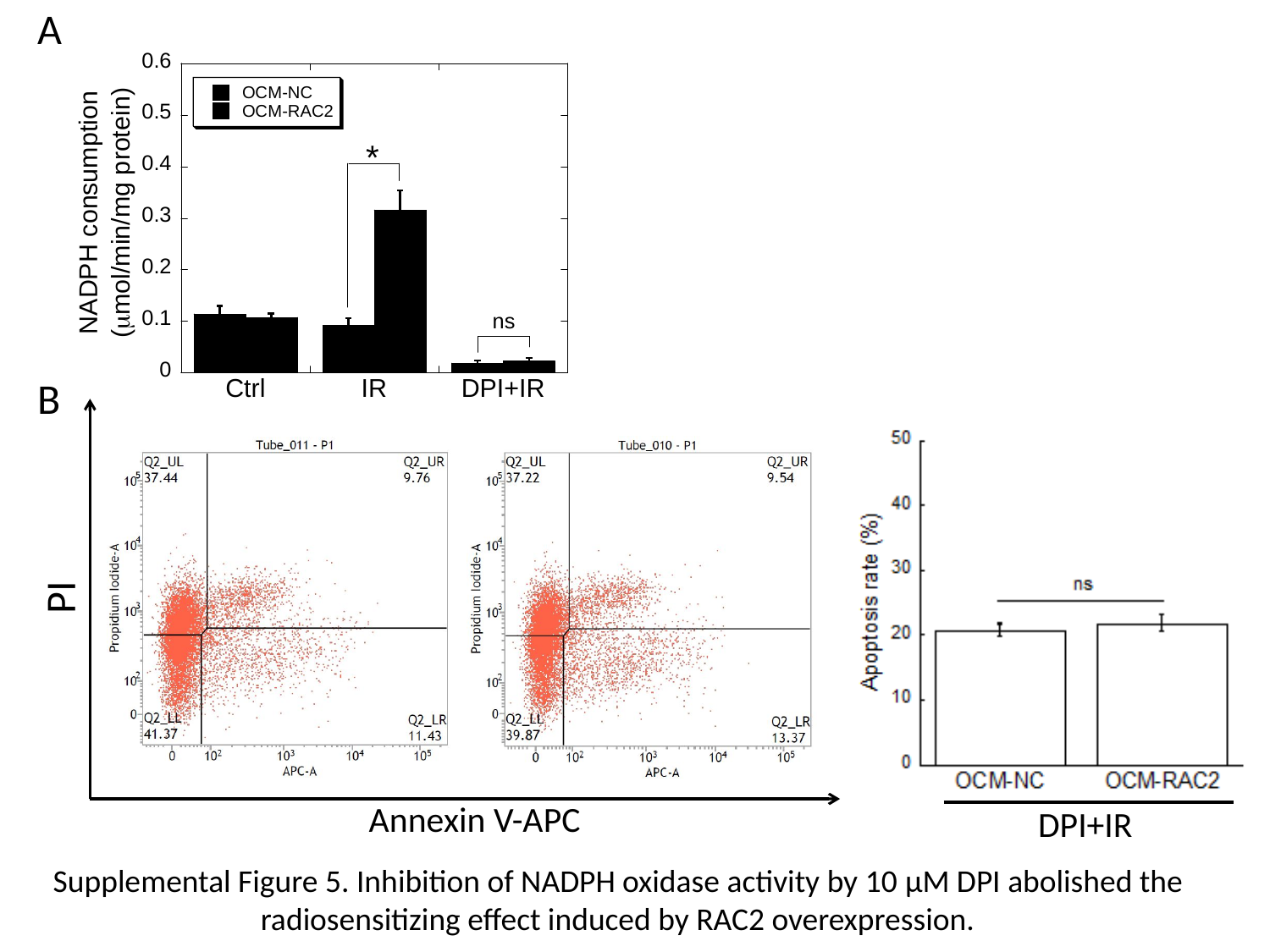

A
B
PI
Annexin V-APC
DPI+IR
Supplemental Figure 5. Inhibition of NADPH oxidase activity by 10 μM DPI abolished the radiosensitizing effect induced by RAC2 overexpression.

## Slide 6
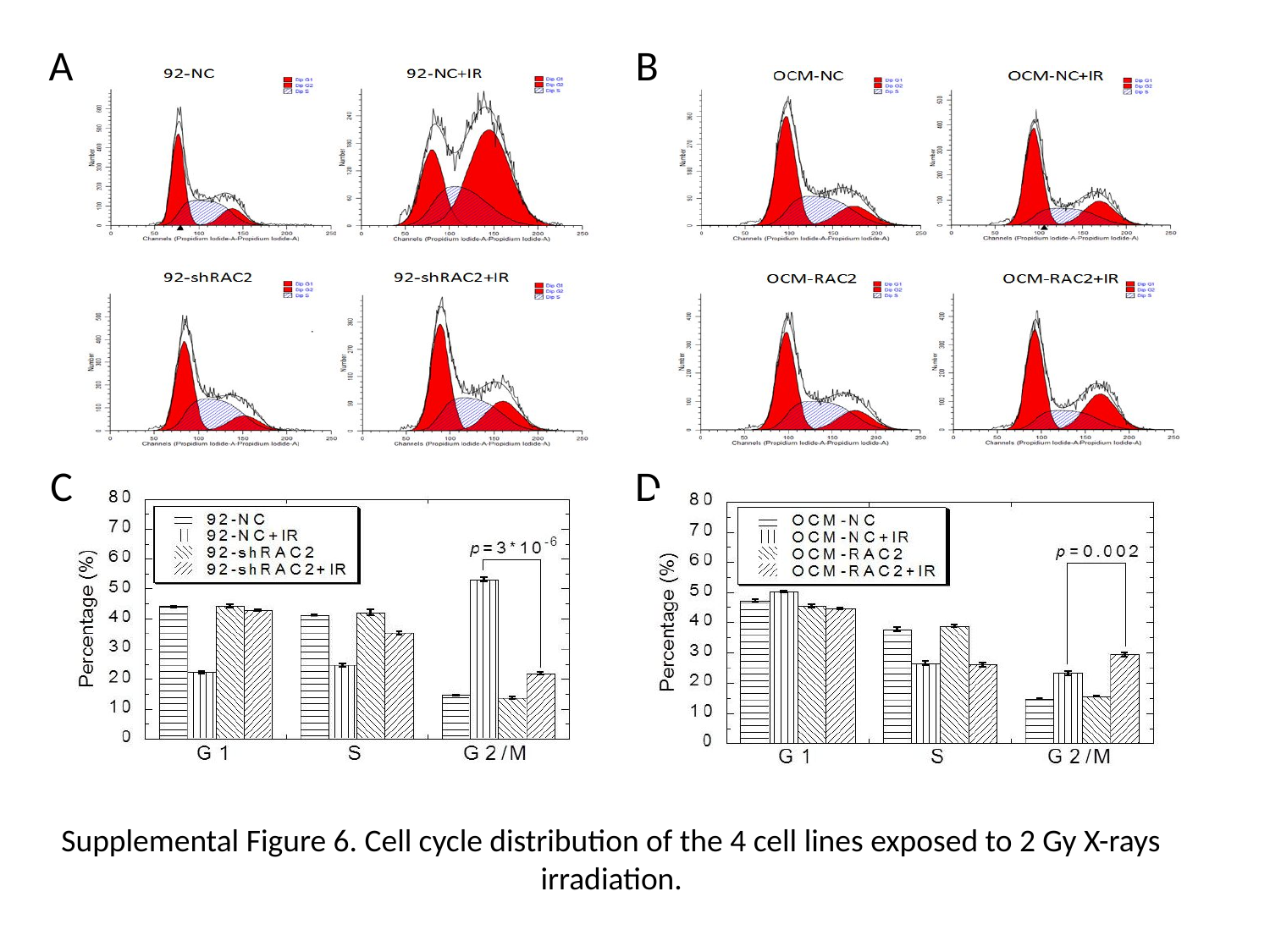

A B
C D
Supplemental Figure 6. Cell cycle distribution of the 4 cell lines exposed to 2 Gy X-rays irradiation.
